# Prevalence and competing risk of psychiatric comorbidities in chronic low back pain by age group

**DOI:** 10.1097/PR9.0000000000001480

**Published:** 2026-07-10

**Authors:** Ryan J. Pontiff, Abigail Helm, Sadaf Arefi Milani, Melissa Morrow, Carole A. Tucker

**Affiliations:** a Department of Physical Therapy and Rehabilitation Sciences, University of Texas Medical Branch, Galveston, TX, USA; b Division of Health Systems Science, Department of Medicine, UMass Chan Medical School, Worchester, MA, USA; c Department of Epidemiology, University of Texas Medical Branch, Galveston, TX, USA

**Keywords:** Chronic low back pain, Prevalence, Competing risk, Psychiatric comorbidity, Age stratification

## Abstract

**Introduction:**

Nonspecific chronic low back pain (cLBP) frequently accompanies psychiatric comorbidities, including anxiety, depression, insomnia, and substance use disorders (SUDs), which worsen patient outcomes. However, research characterizing the prevalence and temporal risk of these comorbidities using large-scale, age-stratified data remains limited.

**Objectives:**

This study evaluated the prevalence of anxiety, depression, insomnia, and SUDs after cLBP diagnosis and the population’s vulnerability to these conditions.

**Methods:**

We performed a retrospective cohort study using the TriNetX network, analyzing deidentified electronic health records from over 150 million patients. Individuals with cLBP from 2014 to 2023 were categorized into age groups (0–25, 26–49, and 50+ years) based on neurodevelopmental milestones and cLBP epidemiology. Annual point prevalence of anxiety, depression, insomnia, and SUDs was calculated for each group. Competing risk analysis determined the 10-year cumulative incidence of each outcome after cLBP diagnosis.

**Results:**

Among 1,045,921 individuals with cLBP, all psychiatric comorbidities rose in prevalence across age groups from 2014 to 2023. By 2023, anxiety prevalence reached 30.8% (0–25), 39.0% (26–49), and 33.0% (50+); depression prevalence was 20.9%, 28.7%, and 27.1%, respectively. Insomnia prevalence increased from 6.9% (0–25) to 13.6% (26–49) and 20.1% (50+). SUD substantially increased from 0 to 25 (7.73%) to 26 to 49 (27.7%) and 50+ (23.2%) groups. For all groups, females showed higher anxiety, depression, and insomnia rates, whereas SUDs were more common in males. Ten-year competing risk cumulative incidence showed that anxiety had the highest risk across all groups: 30.5% (0–25), 27.3% (26–49), 20.5% (50+).

**Conclusion:**

Individuals with cLBP face a rising and substantial burden of psychiatric comorbidities, with age- and sex-specific increasing patterns. Screening and interventions should be tailored by age to improve outcomes for this high-risk population.

## Abstract 2

Patients with nonspecific chronic low back pain increasingly develop anxiety, depression, insomnia, and substance use disorders, with patterns varying by age and sex requiring tailored screening.

## 1. Introduction

Chronic pain, defined as persistent or recurrent discomfort lasting beyond 3 months, affects approximately 24% of US adults and represents one of the most significant public health challenges in modern health care.^28,35^ Nearly 8.5% of Americans experience high-impact pain, characterized by substantial restrictions in daily living activities.^28,35^ Beyond its physiological basis, chronic pain is a complex biopsychosocial phenomenon that is among the most common reasons adults seek medical care and a major risk factor for opioid misuse, anxiety, and depression.^10,29,39,42^ Among chronic pain conditions, low back pain (LBP) is particularly prevalent, affecting an estimated 619 million individuals globally and ranking as the leading cause of years lived with disability in 13 world regions.^8^ Although most LBP episodes resolve naturally, approximately 1 in 5 individuals develops chronic LBP (cLBP),^34^ resulting in increased health care costs and significant reductions in quality of life and work capacity.^13,34^

The biopsychosocial model highlights the complexity of chronic pain through its multifaceted nature as a personal experience that both influences and is influenced by biological, psychological, and social environments, which may benefit from a multidisciplinary treatment approach.^24^ Lived experiences with pain and personal factors such as education, general health, and socioeconomic status shape an individual's learned concept of pain. This complexity becomes particularly evident when chronic pain transitions from its original protective function in acute states to a maladaptive chronic condition involving significant adaptations that may result in heightened sensitivity to pain stimuli. These changes and increased sensitivity can influence cognitive and emotional changes as pain-processing regions become interconnected with areas governing mood, memory, and decision making.^33^ The biological and affective changes may preclude or often codevelop along with mental health disorders in those with conditions such as LBP.^27^

Chronic pain frequently coexists with psychiatric comorbidities, including anxiety, depression, insomnia, and SUDs, creating synergistic relationships that exacerbate disability and complicate treatment.^36^ Among individuals with both chronic pain and opioid use disorder, 91% experience anxiety disorders, 57% experience mood disorders such as depression, and 78% have nonopioid SUDs.^3^ These comorbidities amplify pain perception through shared neurobiological mechanisms, including dysregulated hypothalamic–pituitary–adrenal axis (HPA) axis activity and glutamate/NMDA pathway hyperactivity, as well as maladaptive behaviors such as avoidance and catastrophizing.^25,30^ Together, these factors create a self-perpetuating cycle in which mental health difficulties and pain amplify nociceptive signaling, progressively impairing emotional regulation and coping capacity.^4^

Sleep disturbances, particularly insomnia, are both a consequence and contributor to pain-related disability in cLBP. Among individuals with chronic pain, 23% experience at least 1 insomnia event, whereas 40% of those with insomnia report at least 1 chronic pain condition.^30^ Poor sleep quality and short sleep duration worsen pain sensitivity and increase the risk of pain chronification.^16,32^ The relationship between pain, sleep, and mental health is bidirectional, with anxiety and depression strongly associated with the onset and persistence of sleep disturbances in chronic pain populations. Experimental sleep restriction of 4 hours in rheumatoid arthritis patients significantly increased self-reported pain, fatigue, depression, and anxiety.^20,21^ In addition, individuals with insomnia may misuse substances such as alcohol or cannabis to self-medicate, negatively affecting wakefulness, sensory perception, and judgment, whereas SUDs used for pain or sleep management worsen sleep architecture and independently increase the risk of insomnia and poorer functional outcomes.^26^

Age emerges as a critical modifier in the experience and presentation of these interconnected conditions, as distinct patterns appear in the literature across young, middle-aged, and older adult populations. For example, adults between the ages of 65 and 75 may demonstrate higher pain acceptance and pain self-efficacy, they show lower acceptance of depression and experience higher rates of anxiety.^31,37^ Older adults frequently experience concurrent chronic pain, anxiety, depression, and insomnia,^2^ with insomnia prevalence increasing with age and affecting up to 50% of those with chronic pain.^12^ In older individuals, the next-day impacts of poor sleep on mood, pain, and cognitive performance differ significantly for those with chronic pain compared with those without.^9^ Notably, older adults receiving medication for opioid use disorder report higher pain severity and interference compared with younger adults, with stronger associations with depression and anxiety symptoms, whereas younger adults demonstrate stronger associations between substance use and mental health comorbidities.^46^ For children and adolescents with chronic pain, the risk of developing mental health comorbidities (eg, depression, anxiety) in adulthood is greater compared with those without chronic pain.^15,39,45^

Despite the well-established connections between chronic pain, mental health conditions, sleep issues, and SUDs, significant gaps remain in our understanding of the risk of development and their prevalence patterns across different age groups. Although existing research has begun to identify neurobiological mechanisms linking chronic pain with individual psychiatric conditions, comprehensive epidemiologic analyses that delineate both the co-occurrence of multiple psychiatric comorbidities in cLBP and their joint associations with pain management strategies and patient outcomes remain underexplored.

Current literature lacks examination of robust epidemiological data for the temporal trends and age-specific prevalence patterns of these interconnected conditions in cLBP populations. Furthermore, the competing risks of developing multiple psychiatric comorbidities after chronic pain diagnosis, particularly how age influences these risks, remains poorly understood, limiting our ability to develop targeted prevention and intervention strategies.

Despite receiving care, a substantial proportion of individuals with cLBP continue to report pain, disability, and reduced quality of life. Compared with those without cLBP, population-based samples show that individuals with cLBP have significantly poorer physical function, greater limitations in daily and social activities, more depressive symptoms, and lower health-related quality of life.^17^ We hypothesize that among individuals with cLBP and no prior history of anxiety, depression, insomnia, or SUD, the prevalence of these conditions will increase over time after cLBP diagnosis, as ongoing pain and physical limitations contribute to psychological distress and sleep problems.^43^ Furthermore, we hypothesize that earlier-onset cLBP confers greater long-term vulnerability to anxiety and depression, consistent with evidence that chronic pain diagnosed earlier in life is associated with higher risk of subsequent mental health conditions.

## 2. Materials and methods

This retrospective cohort study used the TriNetX US Collaborative Network, which pools deidentified, standardized electronic health record data from more than 60 anonymized US health care organizations into a single federated network, to obtain a view of the impact of LBP on mental health within the United States. In TriNetX, prevalence measures report all cases recorded within the time frame while competing risks assess a patient's likelihood of experiencing the outcome of interest when combined with other potential outcomes.^5,6^ TriNetX is a federated network that provides real-time access to deidentified electronic health record (EHR) data from health care organizations. The TriNetX network of health care organizations (HCOs) includes large academic medical centers, community hospitals, and specialty clinics across diverse geographic locations. The network encompasses both inpatient and outpatient care settings, providing a comprehensive view of patient care across different health care environments. The platform provides access to structured EHR data, including patient demographics, diagnoses (using ICD-10 codes), procedures, medications, laboratory values, and clinical visits. All data are deidentified in compliance with the Health Insurance Portability and Accountability Act (HIPAA) Privacy Rule, specifically following the deidentification standard defined in Section §164.514(a). The study was exempted by the local Institutional Review Board # 20-0085.

The study sample was queried on July 14, 2025. Prevalence data were included from January 1, 2014, to December 31, 2023. The study period (2014–2023) was selected to provide a 10-year window to evaluate temporal trends while capturing both pre–COVID-19 and COVID-19 eras, thereby enabling assessment of psychiatric comorbidity patterns in nonspecific cLBP under typical health care conditions as well as during and after the pandemic, when mental health burden and care utilization were altered. Competing risk analysis was conducted on each cohort to determine the risk of developing the outcomes of interest between 1 day and 10 years after the development of nonspecific cLBP. Queries were run on TriNetX to establish those with and without an instance of nonspecific cLBP at a visit between 3 months and 10 years. Three cohorts were established based on age in years: cohort 1 (0–25), cohort 2 (26–49), and cohort 3 (50 +). *In this initial epidemiologic analysis, age categories (0–25, 26–49, 50+ years) were selected* a priori *to enable a high-level characterization of comorbidity patterns across the lifespan.* Age groupings were chosen based on adolescent neurodevelopment continuing until approximately 25 years of age for cohort 1 and the highest prevalence of and greatest disability related to nonspecific cLBP peaks after 50.^7,23^

All queries were developed using Current Procedural Terminology (CPT) and International Classification of Diseases 10th Edition (ICD-10) codes. All queries included males and females with nonspecific cLBP ICD-10 code(s) (M54.50, M54.51, M54.59, M79.1, and/or G89.4), selected through consultation with pain and rehabilitation investigators and review of commonly used codes for nonspecific musculoskeletal cLBP within the TriNetX network. Codes indicating radiculopathy, sciatica, other neuropathic spine conditions, or postsurgical states were deliberately excluded to reduce etiologic heterogeneity and better isolate nonspecific cLBP presentations. Of the 70 potential HCOs, 66 to 68 responded to the query, depending on cohort, and were included in the analysis. Clinical variables were extracted using standardized terminologies including ICD-10 codes for diagnoses. Target outcome measures included anxiety (ICD-10-CM: F06.4, F40, F41, F43.0); depression (ICD-10-CM: F32, F33); and insomnia (ICD-10-CM: F51.0, G47.0); SUDs (ICD-10-CM: F10-F19).

For the prevalence analysis, cohort 1 included 83,856 participants, cohort 2 comprised 394,580, and cohort 3 contained 567,485, totaling 1,045,921 individuals. For the competing risks analysis, cohort 1 included 83,903 participants, cohort 2 comprised 395,955, and cohort 3 contained 569,721, totaling 1,049,579 individuals. Minor variation in cohort sizes between analyses reflects the live nature of the TriNetX repository, in which data are updated continuously and each analysis must be calculated separately.

### 2.1. Data analysis

All analyses were conducted using the TriNetX Analytics platform, which uses R statistical software for survival analysis calculations. Point prevalence was calculated as the number of patients with the condition of interest at a specific time point divided by the total number of patients at risk and was analyzed from 2014 to 2023 stratified by age group (0–25, 26–49, and 50+ years) for anxiety, depression, insomnia, and substance use disorder.

To evaluate outcome risk, a competing risks analysis was conducted using the TriNetX platform. Competing risks analysis accounts for events that alter the probability of the primary outcome, providing a more accurate estimate of absolute risk and timing when multiple mutually exclusive outcomes are possible. Participants were classified based on which outcome they were diagnosed with first and subsequently removed from further analysis. The cumulative incidence of anxiety, depression, insomnia, and SUD within 10 years of cLBP diagnosis was evaluated across all 3 age cohorts, including both the percentage of each cohort developing each outcome and the cumulative incidence at the end of the 10-year window.

## 3. Results

### 3.1. Patient demographics

Demographic information for competing risk analysis may be found in Table 1. Across all 3 cohorts, participants were majority female (cohort 1: 55.1%; cohort 2: 53.8%; cohort 3: 55.3%). Racial composition was similarly consistent, with White participants comprising the largest group in each cohort (59.2%, 59.1%, and 68.4%, respectively), followed by Black or African American participants (16.8%, 18.3%, and 13.4%, respectively).

**Table 1:** Demographic information for competing risks.

Competing risk analysis	0–25	26–49	50+
Patients with criteria	377,005	1,261,681	1,574,718
Total records	31,472,281	40,359,477	59,059,028
Health care organizations returning data	70/71	70/71	70/71
Sex			
Male	161,734 (43.2%)	551,747 (43.7%)	643,263 (40.9%)
Female	207,814 (55.1%)	678,710 (53.8%)	870,458 (55.3%)
Unknown	6,457 (1.7%)	31,224 (2.5%)	60,997 (3.8%)
Ethnicity			
Not Hispanic or Latino	226,682 (60.1%)	799,849 (63.4%)	1,0149,078 (66.6%)
Unknown ethnicity	87,932 (23.3%)	313,847 (24.9%)	428,412 (24.2%)
Hispanic or Latino	62,391 (16.6%)	147,985 (11.7%)	97,228 (6.2%)
Race			
White	222,995 (59.2%)	745,989 (59.1%)	1076,659 (68.4%)
Black	63,417 (16.8%)	230,822 (18.3%)	210,232 (13.4%)
Unknown race	42,729 (11.3%)	131,902 (10.5%)	152,579 (9.7%)
Asian	12,214 (3.24%)	50,661 (4.0%)	51,633 (3.3%)
Native Hawaiian	3,266 (0.9%)	10,300 (0.8%)	12,482 (0.8%)
Other	32,384 (8.56%)	92,007 (7.3%)	71,133 (4.4%)

**Figure 1.**
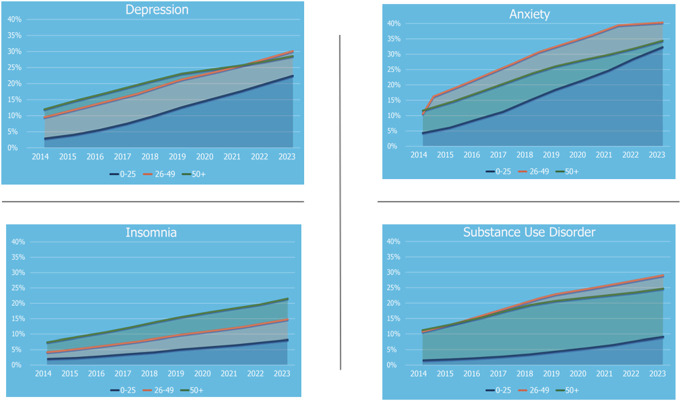
Temporal representation of the increase of each diagnosis over 2014 to 2023.

Figure 1 represents the temporal representation of the increase of each diagnosis over the 2014 to 2023 period.

### 3.2. Prevalence

#### 3.2.1. Anxiety

Anxiety was the most common psychiatric outcome across all age groups. In the youngest cohort (ages 0–25), 16.6% developed anxiety, with a cumulative incidence of 34.2% at 10 years after pain diagnoses. Among those aged 26 to 49, 16.3% developed anxiety, with a cumulative incidence of 30.7%. In the oldest group (50+), 13.2% developed anxiety, and the cumulative incidence reached 16.1% over the same period.

#### 3.2.2. Depression

Depression was less common than anxiety across all age groups. In the youngest cohort (ages 0–25), 4.5% developed depression, with a cumulative incidence of 10.2% at 10 years after pain diagnoses. Among those aged 26 to 49, 5.4% developed depression, with a cumulative incidence of 9.8%. In the oldest group (50+), 6.9% developed depression, and the cumulative incidence reached 10.9% over the same period.

#### 3.2.3. Insomnia

Insomnia was less common than anxiety and depression across all age groups but increased in frequency with age. In the youngest cohort (ages 0–25), 1.7% developed insomnia, with a cumulative incidence of 4.0% at 10 years after pain diagnoses. Among those aged 26 to 49, at baseline, 3.3% developed insomnia, with a cumulative incidence of 6.6%. In the oldest group (50+), 6.6% developed insomnia, and the cumulative incidence reached 11.5% over the same period.

#### 3.2.4. Substance use disorder

Substance use disorder was relatively uncommon in the youngest group but more frequent in older age groups. In the youngest cohort (ages 0–25), 3.2% developed SUD, with a cumulative incidence of 8.9% at 10 years after pain diagnoses. Among those aged 26 to 49, 9.9% developed SUD, with a cumulative incidence of 19.4%. In the oldest group (50+), 9.4% developed SUD, and the cumulative incidence reached 16.1% over the same period.

### 3.3. Competing risks

#### 3.3.1. Anxiety

Anxiety was the most common psychiatric outcome across all age groups. In the 0 to 25 cohort, 16.6% were diagnosed at baseline, with a cumulative incidence of 34.2% at 10 years. In the 26 to 49 cohort, 16.3% were diagnosed at baseline, with a cumulative incidence of 30.7%. In the 50+ cohort, 13.2% were diagnosed at baseline, with a cumulative incidence of 16.1% at 10 years.

#### 3.3.2. Depression

Depression was less prevalent than anxiety across all cohorts. In the 0 to 25 cohort, 4.5% were diagnosed at baseline, with a cumulative incidence of 10.2% at 10 years. In the 26 to 49 cohort, 5.4% were diagnosed at baseline, with a cumulative incidence of 9.8%. In the 50+ cohort, 6.9% were diagnosed at baseline, with a cumulative incidence of 10.9% at 10 years.

#### 3.3.3. Insomnia

Baseline prevalence and cumulative incidence of insomnia increased with age. In the 0 to 25 cohort, 1.7% were diagnosed at baseline, with a cumulative incidence of 4.0% at 10 years. In the 26 to 49 cohort, 3.3% were diagnosed at baseline, with a cumulative incidence of 6.6%. In the 50+ cohort, 6.56% were diagnosed at baseline, with a cumulative incidence of 11.5% at 10 years.

#### 3.3.4. Substance use disorder

Substance use disorder prevalence was lowest in the youngest cohort and higher in older groups. In the 0 to 25 cohort, 3.2% were diagnosed at baseline, with a cumulative incidence of 8.9% at 10 years. In the 26 to 49 cohort, 9.9% were diagnosed at baseline, with a cumulative incidence of 19.4%. In the 50+ cohort*, 9.4% were diagnosed at baseline, with a cumulative incidence of 16.1% at 10 years.*

## 4. Discussion

This study using the TriNetX platform provides important insights into the prevalence and burden of psychiatric comorbidities in cLBP across age groups. Anxiety was the most frequent psychiatric outcome across all cohorts, with the highest cumulative incidence in younger individuals and progressively lower rates in older groups. Depression and insomnia were less prevalent but increased consistently over time, with depression slightly elevated in older adults and insomnia showing a clear age-related upward gradient. SUD was least common in the youngest cohort but substantially more prevalent in middle-aged and older adults, highlighting important age-related differences in psychiatric comorbidity profiles.

Across age groups (0–25, 26–49, and 50+ years), the racial makeup of our cLBP group closely resembled current US distributions based on the 2022 American Community Survey (ACS) 5-year DP05 profile. In each age category, White participants made up about 59% to 71% of the sample, Black participants 15% to 20%, and Asian participants 3% to 4%, with smaller shares of American Indian, Native Hawaiian/Other Pacific Islander, and other races, aligning with national ACS estimates for these groups, in which the 2022 ACS reports the US population as 65.9% White, 12.5% Black, and 5.8% Asian. This consistency with ACS 5-year demographic data supports the broader applicability of our findings to major racial groups among US adults with cLBP.^44^

Prevalence rates of anxiety, depression, insomnia, and SUDs increased consistently across all age groups from 2014 to 2023, aligning with broader epidemiological trends documenting rising mental health conditions in the general population from 2009 to 2019.^14^ A systematic review and meta-analysis by Aaron et al. found that approximately 40% of adults with chronic pain experience clinically significant depression and anxiety,^1^ consistent with the elevated prevalence rates observed in this study, particularly in later years of the observation period. The steepest increases in the current study occurred after 2019, coinciding with the COVID-19 pandemic period, which was associated with significant deterioration in mental health outcomes and significantly higher cumulative incidence of both depression and anxiety among individuals with cLBP compared with those without.^22,38^ Korean studies have documented that COVID-19 led to decreased exercise duration, reduced sleep quality, and increased depression among chronic pain patients, factors that were significantly associated with worsening pain.^22^

Age-related differences in prevalence and competing risks outcomes reveal clinically significant patterns. The highest burden of anxiety and depression was consistently observed in the 26 to 49 age group across both analyses, contrasting with prior research suggesting older adults experience higher rates of psychiatric comorbidity with chronic pain.^31^ This discrepancy may partly reflect differences in age classification, as Murry et al. defined middle-aged as 40 to 65 and older adults as 65 to 75, whereas this study classified older adults as 50+ based on increased pain rates after this age.^7,31^ Our findings align with meta-analytic evidence indicating that anxiety and depression more strongly correlate with functional outcomes than pain intensity in younger adults.^18^ Insomnia prevalence increased with age, consistent with longitudinal research demonstrating that chronic musculoskeletal pain prospectively predicts insomnia in older adults.^40^

In this framework, the cumulative incidence functions estimate the probability that a given psychiatric diagnosis is the first recorded diagnosis after cLBP, in the presence of other potential diagnoses. These estimates, therefore, reflect the ordering of recorded diagnoses in EHR data, not the full clinical history or co-occurring symptomatology. The competing risks analysis provides novel insights into which psychiatric comorbidities after cLBP diagnosis may be first recorded. Anxiety emerged as the most frequent outcome across all age groups, ranging between 20% and 30%, over 10 years of follow-up. These findings are consistent with conceptual models in which anxiety frequently emerges early in the course of chronic pain and is closely linked to subsequent depressive symptoms; however, our competing-risk framework and EHR-based data cannot establish the true clinical ordering or causal pathways among these conditions.^38^ The relatively lower cumulative incidence of depression after excluding those first diagnosed with anxiety indicates that depression is less often the first *recorded* psychiatric diagnosis than anxiety in this dataset and should not be interpreted as evidence that anxiety causally or uniformly precedes depression.

In our analysis, SUD represents an aggregated construct encompassing alcohol-, opioid-, cannabis-, stimulant-, and other substance-related diagnoses, which differ in their epidemiology and clinical implications in chronic pain populations. Substance use disorder patterns revealed important age-related variations, with SUD being notably more prevalent in the middle-aged (26–49) and older (50+) cohorts compared with the youngest group in the competing risks analysis. This pattern aligns with literature documenting that approximately 65% of patients with SUDs experience chronic pain, of which 61.8% had chronic pain before the SUD.^19^ In addition, back pain has been found to be positively associated with SUD, with SUD being more common in men than in women.^41^ In a qualitative study by Wyse et al., 3 primary pathways linked chronic pain and substance use: independent development, self-medication of pain, and opioid-medication–initiated addiction.^47^ This finding highlights the clinical importance of recognizing that individuals with chronic pain may develop SUD through diverse pathways. Given this heterogeneity and the combined nature of our SUD measure, our results should be seen as emphasizing the overall burden of substance-related comorbidity in chronic pain rather than specific substance mechanisms. This highlights the importance of integrated, multidisciplinary approaches for managing pain and mental health.

The sex differences observed in this study among individuals with SUD and chronic pain, with females consistently showing higher rates of anxiety, depression, and insomnia and males showing higher rates of SUD, are consistent with prior literature.^11,29^ A systematic review of sex differences in comorbid pain and opioid use disorder found that women typically report greater psychiatric comorbidity and physical impairment from pain, whereas men report more aberrant or abusive tendencies when exhibiting substance use behaviors.^29^ These descriptive findings are hypothesis-generating and should be interpreted cautiously, as they may reflect differences in health care utilization, prescribing patterns, and diagnostic practices as well as underlying clinical factors; future work is needed to determine whether and how sex-informed approaches to assessment and treatment could improve outcomes.^29^

These findings have important clinical implications for cLBP management. The high cumulative incidence of psychiatric comorbidities, particularly anxiety, underscores the need for routine screening and early intervention across all age groups. Age-specific patterns further suggest that tailored approaches are warranted, with emphasis on comprehensive mental health assessment in younger adults (0–25 and 26–49), who demonstrated the highest rates of anxiety and depression. In addition, the temporal trends highlighting dramatic increases after 2019 emphasize the potential impact of societal stressors on mental health outcomes in chronic pain populations. Thus, health care providers should be vigilant about assessment of psychiatric comorbidities during periods of heightened personal and societal stress and consider the broader psychosocial context when developing treatment plans.

From a public health perspective, these findings highlight the substantial burden of psychiatric comorbidity in cLBP populations, particularly the dramatic increase in anxiety prevalence in the 26 to 49 age group, reaching nearly 40% by 2023. Approximately 12 million US adults experience co-occurring chronic pain and anxiety or depression, with individuals with chronic pain being approximately 5 times more likely to report anxiety or depression compared with those without.^10^ The competing risks analysis further underscores this burden, demonstrating that the 10-year risk of developing at least 1 psychiatric comorbidity after cLBP diagnosis is substantial, with important implications for health care resource allocation and the development of integrated care models addressing both pain and mental health simultaneously.

Several limitations should be acknowledged. Reliance on EHR data introduces potential biases related to coding accuracy and completeness, and the database primarily captures patients who seek medical care, limiting the representativeness of those who do not, which is a well-established limitation of TriNetX. The competing risks analysis assumes that development of 1 psychiatric condition precludes others within the analytical timeframe, which may not reflect clinical reality where multiple comorbidities frequently co-occur. As TriNetX aggregates data predominantly from academic medical centers and acute care institutions, the sample is subject to selection bias toward insured populations, limiting generalizability. Unmeasured confounders, including socioeconomic status, pain severity, and treatment characteristics, may have influenced results. The observational design precludes causal inference between cLBP and psychiatric outcomes. Given the frequent co-occurrence of anxiety, depression, insomnia, and SUD, and the potential for delayed or differential EHR coding, competing risk estimates should be interpreted as probabilities of first recorded diagnosis rather than mutually exclusive psychiatric trajectories. Finally, broad age categories and aggregated ICD-based constructs may obscure differences between more specific subgroups, warranting greater granularity in future analyses.

## 5. Future directions

Future research should prioritize integrated, age- and sex-specific interventions targeting cLBP and psychiatric comorbidities, alongside longitudinal studies to clarify causal pathways and identify modifiable risk factors. The post-2019 rise in psychiatric comorbidities warrants investigation into societal stressors and resilience-building strategies. Studies should also incorporate socioeconomic variables, pain characteristics, and diagnostic subtypes to advance mechanistic understanding and support personalized treatment approaches.

## 6. Conclusion

This study provides compelling evidence for the high and increasing burden of psychiatric comorbidities in cLBP populations, with important age-related variations that have significant implications for clinical practice and public health policy. The findings underscore the critical need for integrated, age-appropriate screening and treatment approaches to address the substantial and growing burden of psychiatric comorbidity in those with cLBP.

## Disclosures

The authors have no conflict of interest to declare.
